# Threshold voltage decrease in a thermotropic nematic liquid crystal doped with graphene oxide flakes

**DOI:** 10.3762/bjnano.10.7

**Published:** 2019-01-07

**Authors:** Mateusz Mrukiewicz, Krystian Kowiorski, Paweł Perkowski, Rafał Mazur, Małgorzata Djas

**Affiliations:** 1 Institute of Applied Physics, Military University of Technology, 00-908 Warsaw, Poland; 2 Department of Chemical Synthesis and Flake Graphene, Institute of Electronic Materials Technology, 01-919 Warsaw, Poland

**Keywords:** graphene oxide, liquid crystal, nematic phase, switching, threshold voltage

## Abstract

We report a threshold voltage decrease in a nematic liquid crystal compound, 4-cyano-4′-pentylbiphenyl (5CB), doped with graphene oxide (GO) flakes at a concentration of 0.05–0.3 wt %. The threshold voltage decrease was observed at the same concentration in electro-optic and dielectric spectroscopy measurements. The effect is related to the disrupted planar alignment due to the strong π–π stacking between the 5CB’s benzene rings and the graphene oxide’s structure. Additionally, we present the GO concentration dependence on the isotropic–nematic phase transition temperature, electric anisotropy, splay elastic constant, switch-on time, and switch-off time. The shape and dimensions of the GO flakes were studied using atomic force microscopy (AFM) and scanning electron microscopy (SEM). The influence of the GO concentration on the physical properties and switching process in the presence of the electric field was discussed.

## Introduction

Liquid crystals (LCs) are classified as a type of soft matter which are characterized by anisotropic molecules and a liquid-like fluidity behavior. Of all LC phases, special attention is paid to the nematic liquid crystal (NLC) phase because is widely used in many electro-optical applications [1–2]. In the uniaxial nematic phase, the direction of the optical axis is described by the director ***n***, which is the unit vector along the molecular axis. An applied electric field can change the director orientation thereby causing a change in the optical properties. In the absence of an electric field, the orientation of ***n*** is determined by anchoring conditions. The field-induced reorientation of the LC director is known as the Frédericksz effect [3]. In the Frédericksz effect, the deformation of a homogeneous layer of a NLC is caused by the electric field ***E***, which is initially perpendicular to the director. Such structural transition appears at a certain magnitude called the threshold voltage, *U*_th_. When the applied voltage, *U*, is lower than the threshold *U* < *U*_th_ the tilt angle θ is small. When the voltage is above *U*_th_, we start to observe the increase of θ [4]*.* The complete reorientation of the director ***n***, θ = 90°, occurs at higher voltages *U* > *U*_th_. The reorientation is caused by the anisotropy of the electric permittivity 
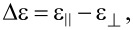
 where ε*_||_* and 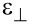 is the electric permittivity measured along the directions parallel and perpendicular to ***n***, respectively. In the case of positive electric anisotropy (Δε > 0), the director is forced to align along the electric field.

One of the most efficient methods to reduce the threshold voltage in nematic liquid crystals for high electro-optical performance is doping them with nanoparticles. Hsu et al. showed that the small addition of gold nanoparticles decreases *U*_th_ due to the increased electric anisotropy and decreased elastic constant [5]. Haraguchi and collaborators doped the twisted nematic liquid crystal cell with inorganic nanoparticles of MgO and SiO_2_ [6]. Here, the effect was caused by the reduction of the order parameter *S*. The concentration-dependent enhancement of the electro-optic response was observed for Ti and TiO_2_ nanoparticles dispersed in NLC [7–8]. The significant effect of ferroelectric Sn_2_P_2_S_6_ on the threshold voltage was reported by Reznikov et al. [9]. Materials with a suspension of ferroelectric, submicrometer particles exhibit a lower threshold voltage and enhanced electric anisotropy compared to pure liquid crystal compounds [9–10].

Extensive research has also been performed on carbon-based material composites [11–14]. NLCs doped with graphene or carbon nanotubes show a faster response in electro-optical switching than pure liquid crystal compounds. This effect is caused by trapping of some free ion concentrations [15–16] or reduction in the rotational viscosity [17]. Furthermore, due to strong π–π electron stacking of liquid crystal molecules on graphene sheets, one obtains pseudo-nematic domains [18], which enhance the electric anisotropy in the nematic phase. Carbon nanotubes doped into the nematic liquid crystal can effectively reduce the driving voltage due to the increase of the elastic constant [19–21].

In this work, we discuss our approach in reducing the threshold voltage by using graphene oxide (GO) flakes. The extraordinary properties of GO make this material a good candidate for this purpose. GO is an oxidized form of graphene [22–24]. In GO, carbon atoms are highly decorated with various oxygen-containing groups, for instance, hydroxyl, carbonyl, carboxyl and epoxy [25]. In contrast to graphene, the oxygen groups make graphene oxide layers hydrophilic [25], negatively charged [23] and insulating. The oxidized layers are randomly distributed with non-oxidized areas. Therefore, the main advantage of GO is easy dispersibility in water and organic solvents [23,25]. Graphene oxide occurs in the form of two-dimensional flakes with anisotropic properties [24]. In thermotropic nematic liquid crystals, GO flakes create dipoles due to Maxwell–Wagner polarization [26]. An applied electric field acts to induced dipole moments, which causes reorientation of the flakes [27–28]. The above-mentioned facts are very important features for mixing a nematic liquid crystal with GO flakes in order to improve its physical properties.

Here, the nematic liquid crystal compound 4-cyano-4′-pentylbiphenyl (5CB) was doped with low concentrations (0.05–0.3 wt %) of GO flakes. We found that using the GO flakes we are able to reduce the threshold voltage in the Frédericksz effect. We report and discuss the isotropic–nematic phase transition temperature, splay elastic constant *K*_11_, electric anisotropy Δε, switch-on τ_ON_ and switch-off τ_OFF_ times and their dependence on the concentration of the GO flakes.

## Materials and Methods

**Figure 1:**
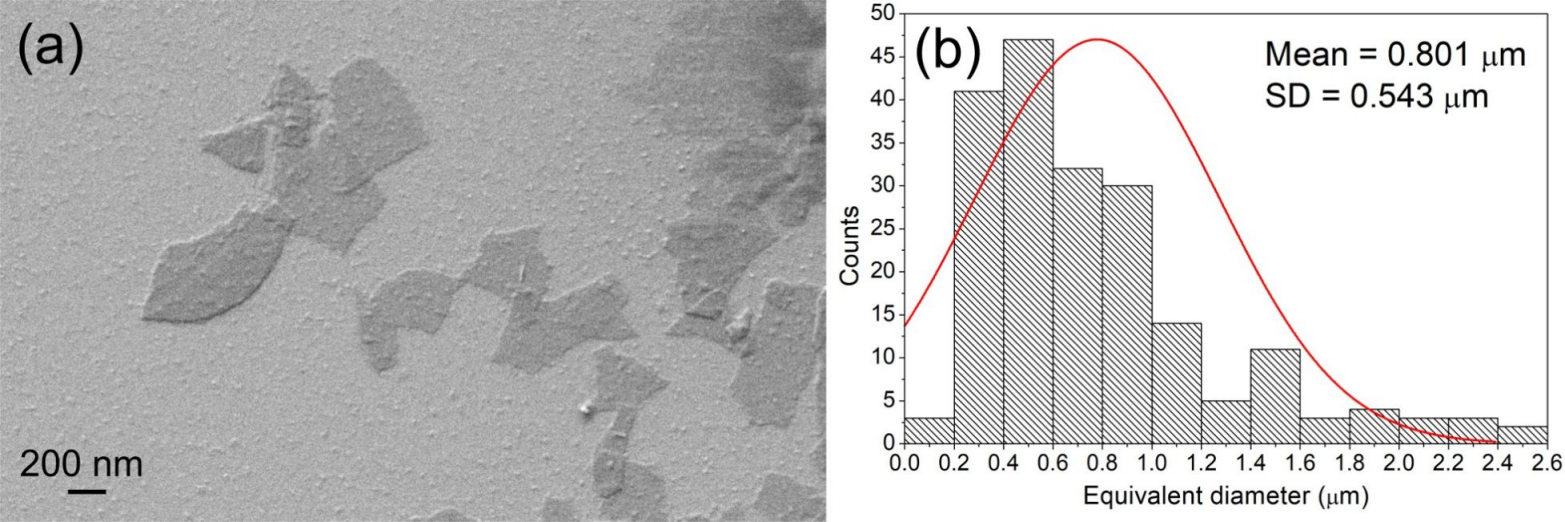
(a) SEM image of graphene oxide flakes, (b) the equivalent diameter distribution of the GO flakes obtained by SEM.

**Figure 2:**
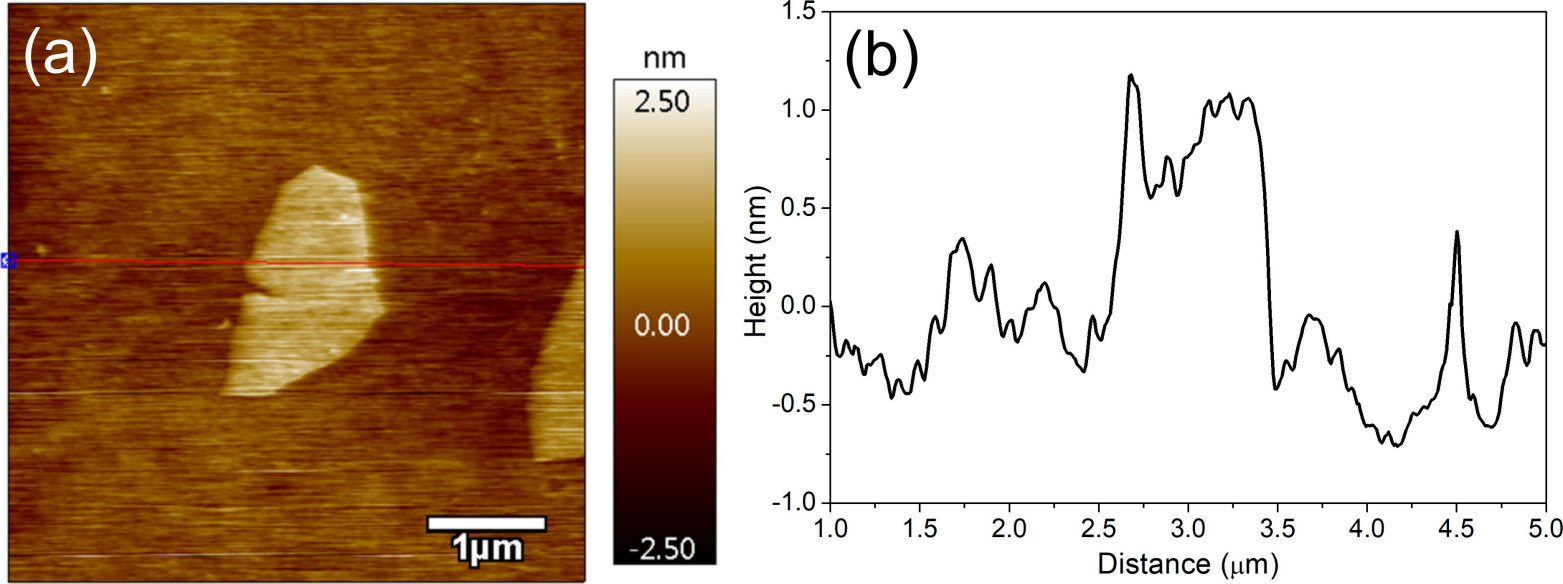
(a) AFM image and (b) AFM height image of the selected GO flake sample.

Graphene oxide (GO) flakes dispersed in water were obtained from natural graphite by the modified Hummers’ method [29–30] in the Department of Chemical Synthesis and Flake Graphene, Institute of Electronic Materials Technology, Warsaw, Poland. Then, the GO flakes in water were transferred by solvent exchange into isopropanol (IPA) and were sonicated to achieve a stable GO-IPA suspension of a known concentration. The structure of the flakes was characterized by scanning electron microscopy (SEM, Auriga Cross Beam Workstation, Carl Zeiss). The GO flakes had an irregular two-dimensional shape (Figure 1a). Additionally, we observed that they exhibit a tendency to fold and wrinkle. In the experiment, the mean equivalent diameter of the GO flakes was 0.801 μm while the standard deviation (SD) was equal to 0.543 μm (Figure 1b). The calculations were done using ImageJ software (V. 1.52a). The thickness of the GO flake samples was measured using a MFP 3D BIO (Asylum Research/Oxford Instruments) atomic force microscope (AFM) working in semi-contact regime (Figure 2a). This technique can provide qualitative and quantitative information about tested samples [31–33]. To register topographical maps, silicon AC240 TS (Olympus) scanning probes were used in a frequency range from 0.8 to 1.0 Hz. The nominal probe spring constant was 2.7 N/m and the radius was <10 nm. The AFM examination was carried out in air, under ambient conditions. The data analysis was conducted with IgorPro (V. 6.17, professional, dedicated software provided by the microscope producer). The measured thickness was around 1–2 nm (Figure 2b), which corresponds to 2–4 single layers of graphene. After characterization by SEM and AFM, the GO-IPA suspension was added to 4-cyano-4′-pentylbiphenyl (5CB) to obtain different GO concentrations (0.05–0.3 wt %). Isopropanol was evaporated at 45 °C for 24 h.

The rod-like 5CB compound was synthesized in the Institute of Chemistry, Military University of Technology, Warsaw, Poland. It is a room temperature nematic liquid crystal material of high chemical stability. The phase transition from the isotropic phase to the nematic phase and then to the crystalline phase is at 35 °C and 18 °C, respectively [34–36]. Pentylcyanobiphenyl (5CB) has a large longitudinal (µ_||_) electric dipole moment, which equals 6.37 D, while the transverse component (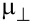) is 0.15 D [36]. Hence, the material shows a relatively large positive electric anisotropy Δε (Δε ≈ 12 at 1 kHz and 25 °C) [34,36–37].

The nematic liquid crystal materials doped with the GO flakes were filled by capillary action into cells. The cells consisted of two pieces of parallel glass plates of thickness *d* = 1.1 mm (Precision Glas & Optik GmbH), covered by thin (*d* ≈ 100 nm) ITO conducting layers, separated by 1.6 µm thick glass spacers. An active area was 25 mm^2^. On the glass plates a polyimide SE-130 (Nissan Chemicals) of *d* ≈ 30 nm was deposed and rubbed to induce planar alignment (HG, homogenous) of the director ***n***. The isotropic–nematic phase transition temperature was determined by means of the transmitted light intensity (TLI) technique, as a function of temperature. The temperature was controlled with a Linkam TMS 93 controller and a heating stage, THMSE 600. The intensity of light propagating through the cell under crossed polarizers, without the applied electric field, was registered with a photodetector, FLC Electronics PIN 20. The switching times were measured using the same experimental set-up. In the electro-optic (EO) experiment, the switching was driven by the ac square electric signal at a frequency of 1 kHz generated by a waveform generator, Agilent 33220A. Moreover, to determine the switching times in the electrically controlled birefringence (ECB) mode of operation, an oscilloscope (Tektronix TDS 2014) was used to register the electro-optical response. The duration of the driving pulse was 1 s. Dielectric spectroscopy (DS) studies were performed using an impedance analyzer (Agilent 4294A) and conducted over a broad frequency range from 100 Hz to 10 MHz, with the oscillation level (ac) at 0.1 V and with different (dc) bias voltages. All measurements were performed at 23 °C.

## Results and Discussion

**Figure 3:**
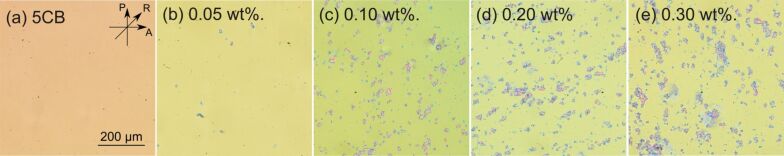
Liquid crystal textures of 5CB doped with graphene oxide flakes, captured at 23 °C. The optical polarizing images were taken in planar aligned cells for: a) the pure 5CB and 5CB-GO suspensions at concentration of b) 0.05 wt %, c) 0.1 wt %, d) 0.2 wt %, and e) 0.3 wt %.

The influence of the GO flakes on the quality of alignment in the planar oriented liquid crystal cells was studied by observation of liquid crystal textures using the polarizing optical microscope (POM) technique. In the all liquid crystal composites, the GO flakes were well dispersed. The liquid crystal cells exhibited uniform color, indicating that the rod-like molecules were uniformly aligned. However, Figure 3b–e, illustrates some GO aggregation. The size and quantity of the aggregates is much more visible for the higher concentration (Figure 3e) than for the lower concentration (Figure 3b). The large aggregates disorder the long-range orientational order and disturb the liquid crystal alignment. According to the classical Michel–Levy interference color chart [38], we observe a slight decrease in birefringence of GO composites compared with the pure 5CB nematic liquid crystal (Figure 3a). For concentrations higher than 0.3 wt %, we can expect the creation of a lyotropic nematic phase in the isotropic phase of the 5CB-GO suspension, as was previously reported [39–40].

**Figure 4:**
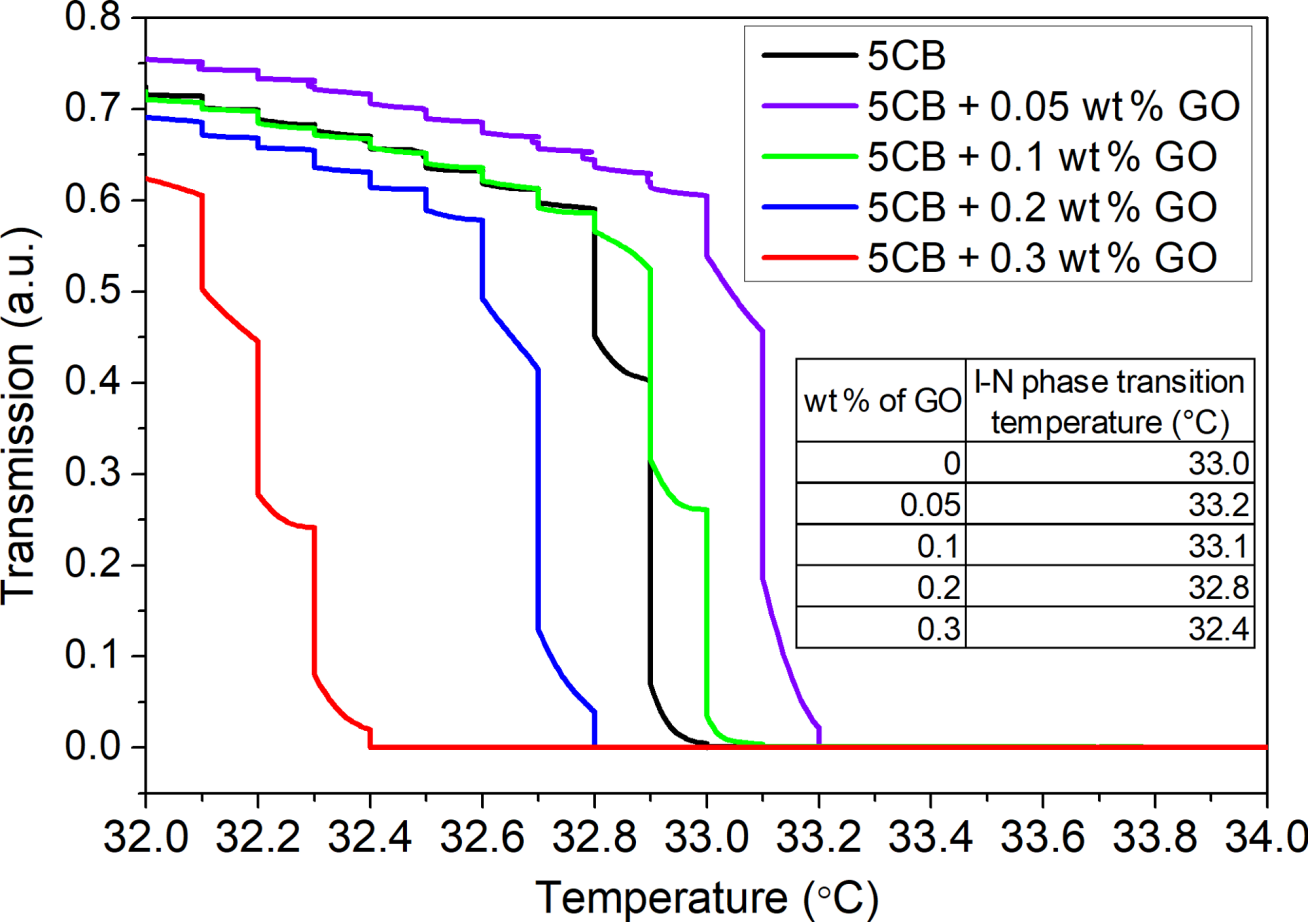
Normalized transmission upon temperature for the pure 5CB and the 5CB-GO composites. The inset shows the clearing temperature dependence on the GO concentration.

We used the TLI technique for making precise measurements of phase transition temperatures. In the case of the isotropic–nematic phase transition, in the isotropic state, the intensity of light propagating through the cell under crossed polarizers is around zero (dark state). During the cooling process, at the phase transition temperature *T*_I-N_, the transmission of light starts to increase due to the nucleation of the nematic phase (bright state). In the experiment, the clearing temperature of the pure 5CB (33.0 °C) was 2 °C below that of previous reports [34,36]. The effect of the lower *T*_I-N_ is related to the strong boundary conditions of the thin cell. As shown in Figure 4, increasing the GO concentration up to 0.1 wt % increases the isotropic–nematic phase transition by about 0.1–0.2 °C. For concentrations higher than 0.1 wt % *T*_I-N_ is shifted towards lower temperatures. For example, in the 5CB-GO suspension of 0.3 wt % the phase transition temperature occurs at 32.4 °C. These results are consistent with the previous studies on the mixture of 5CB and 7CB with the addition of GO [12]. It was reported that the *T*_I-N_ temperature increases at lower concentrations of GO, while it decreases as the ratio is increased above the certain value (0.75 wt %). The difference in phase transition temperatures of the 5CB-GO composites can be attributed to the different interactions between the liquid crystal molecules and the GO flakes. On the one hand, for the lower GO ratios 0.05–0.1 wt % the nematic order is enhanced by the strong π–π stacking. The π–π stacking between the 5CB molecules and the GO flakes causes the appearance of the nematic-like domains in the isotropic phase. Therefore, we observe at higher temperatures the creation of the nematic phase. On the other hand, when the concentration of GO exceeds 0.1 wt %, the GO flakes disturb the formation of the nematic phase.

In this experiment, the Frédericksz transition was induced by the electric field. The threshold voltage dependence on the GO concentration in 5CB was investigated by two methods: an electro-optic (EO) and dielectric spectroscopy (DS) experimental technique. On one hand, in EO we measure the intensity of light passing through the cell between crossed polarizers as a function of an applied voltage. On the other hand, DS is based on measurements of electric permittivity ε at a given frequency under different bias (dc) electric fields. These experimental techniques allowed to obtain two independent characteristics of the threshold voltage. The main difference in the above-mentioned methods is that in the DS measurements we are able to observe a full orientation of the director at *U*_th_. In the case of the EO technique, we detect the beginning of the switching mechanism. A discrepancy is a consequence of the fact that EO measurements are carried out on a small, isolated area while in DS data are collected from a whole ITO electrode. For this reason, the values of the threshold voltage from the DS investigations (*U*_th-DS_) using the bias electric field are overstated compared to the results from the EO experiment (*U*_th-EO_).

**Figure 5:**
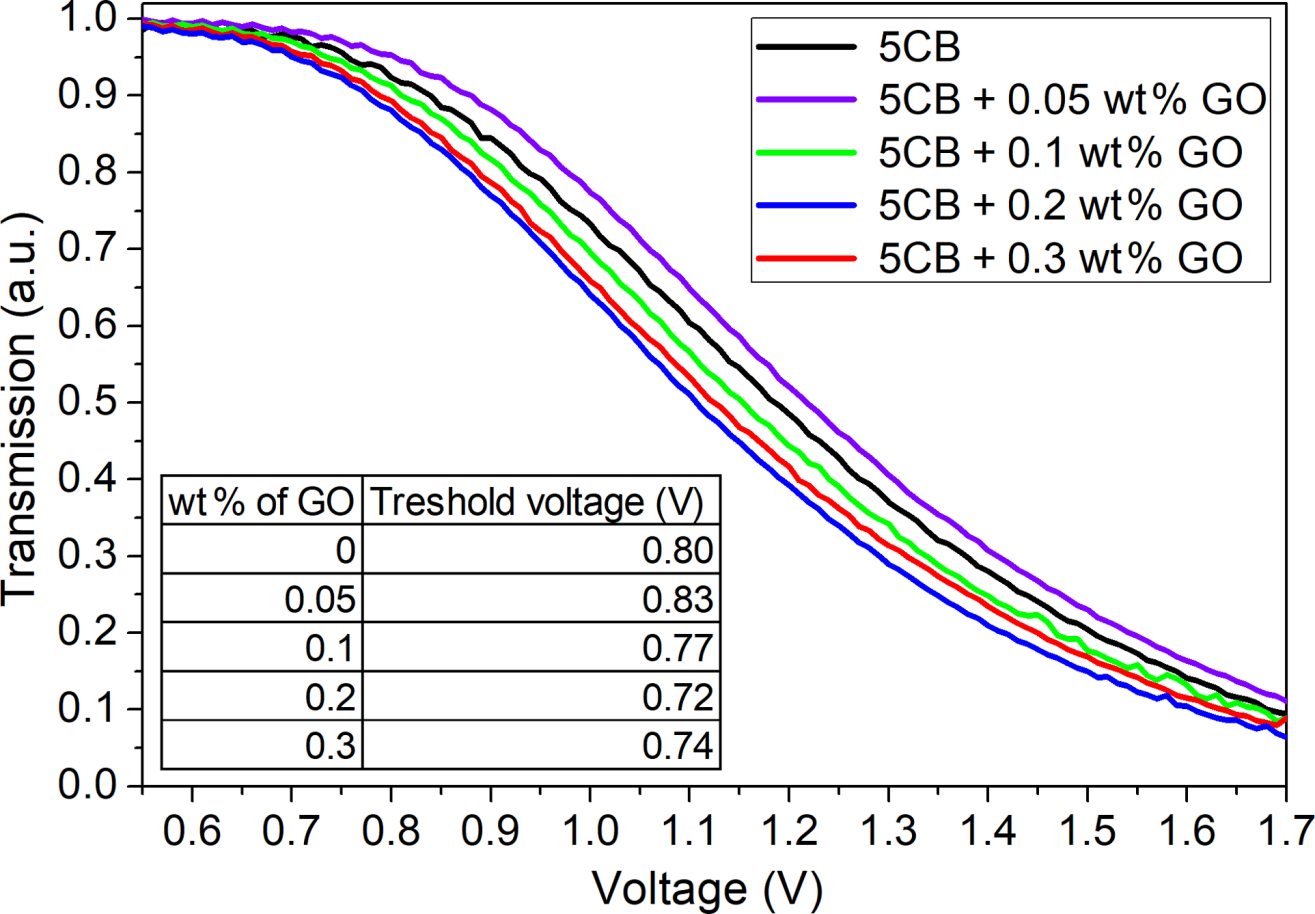
Normalized transmission of light as a function of applied voltage for the pure 5CB and the 5CB-GO suspensions at the different concentrations. The inset shows the threshold voltage *U*_th-EO_ dependence of the GO concentration.

Figure 5 presents the transmission of light versus the applied electric field. The *U*_th-EO_ in EO is defined as the voltage at which the transmission of polarized light starts decreasing. Compared to the pure 5CB, the experimental results show the increase of the threshold voltage at the concentration of 0.05 wt %. However, further increase of the GO ratio results in the opposite trend. We observe the shift of the transmission curves towards the lower voltages. The highest reduction in *U*_th-EO_ is observed at 0.2 wt % of GO, the threshold voltage is reduced by 10% (decrease from 0.80 V to 0.72 V).

**Figure 6:**
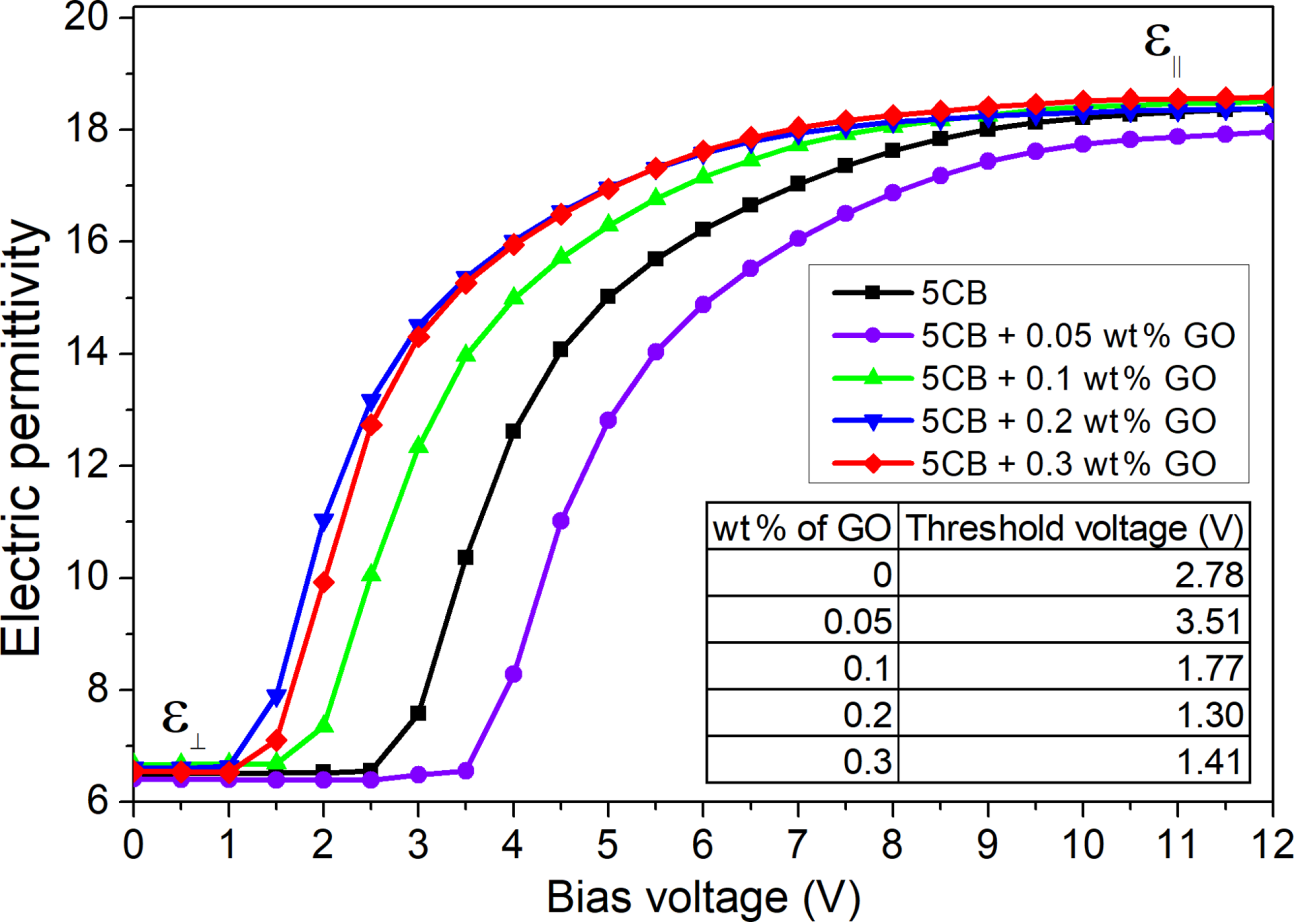
Electric permittivity ε dependence upon the bias (dc) electric field at a frequency of 1 kHz. Results obtained for the pure 5CB and the 5CB-GO suspensions at different concentrations. The inset shows the threshold voltage *U*_th-DS_ dependence of the GO concentration. Experimental data is presented as scattered points with the line as a guide for the eye.

In DS the threshold voltage *U*_th-DS_ is defined as the point where electric permittivity ε starts increasing from the initial value (perpendicular component 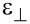 of electric permittivity tensor). Thus, for the high bias voltages, we are able to determine the parallel component ε_||_ from the plateau range of the measured electric permittivity ε. Figure 6 presents the electric permittivity characteristic as a function of the bias (dc) electric field. Here, we observe the decrease of *U*_th-DS_ at the same concentration (0.2 wt %) as in the case for the EO measurements. However, in DS, the effect of the GO flakes on the threshold voltage is much more pronounced. At a concentration of 0.2 wt %, the threshold voltage (*U*_th-DS_ = 1.30 V) is lower by almost half as compared to the pure 5CB (*U*_th-DS_ = 2.78 V). As in the case for EO in DS we observe the increase in the threshold voltage at 0.3 wt %. The obtained values of 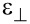 and ε*_||_* in this experiment allow us to calculate the electric anisotropy Δε of the investigated materials. The detailed results are gathered in Table 1. We found that Δε slightly changes in the 5CB-GO composites. The highest value of Δε is observed at 0.20 wt % (Δε = 12.22), while the lowest is at 0.05 wt % (Δε = 11.77), compared to the pure 5CB (Δε = 12.04).

**Table 1:** Values of the electric anisotropy Δε in the 5CB-GO composites as a function of the GO concentration.

wt % of GO	Δε (1 kHz, 25 °C)

0	12.04
0.05	11.77
0.1	11.96
0.2	12.22
0.3	12.05

Knowing the threshold voltage *U*_th-EO_ from the EO measurements and the electric anisotropy Δε of the GO composites one can calculate the elastic constants, *K*_11_ values, from the formula: 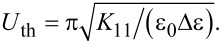 The elastic constant is a parameter characterizing the elastic interaction between the nematic molecules. However, when GO flakes are inserted in the cell, the value of *K*_11_ of the 5CB-GO composites also reflects the interaction between the molecules and the inserted GO flakes [41–42]. In Table 2 we see that the threshold voltage behavior of the 5CB-GO suspensions is reflected in the concentration dependence of *K*_11_. The small amount (0.05 wt %) of the carbon dopant leads to deterioration of properties relevant for liquid crystal applications. This is due to the increase of *U*_th_ and *K*_11_ and the decrease of Δε. The low concentration of GO flakes stiffen the structure of the 5CB-GO composites. For this reason, the reorientation of ***n*** is more difficult. However, at concentration of 0.2 wt % the material parameters are improved. As the concentration of GO flakes increases, the structure starts to be less uniform than in pure 5CB and a large number of defects makes this composite easier to reorient. Moreover, the director field is disturbed by aggregation of GO flakes.

**Table 2:** Threshold voltage *U*_th-EO_ and the calculated results of the splay elastic constants *K*_11_ in 5CB-GO composites as a function of GO concentration.

wt % of GO	*U*_th-EO_ [V]	*K*_11_ [pN]

0	0.80	6.83
0.05	0.83	7.26
0.1	0.77	6.30
0.2	0.72	5.71
0.3	0.74	5.92

**Figure 7:**
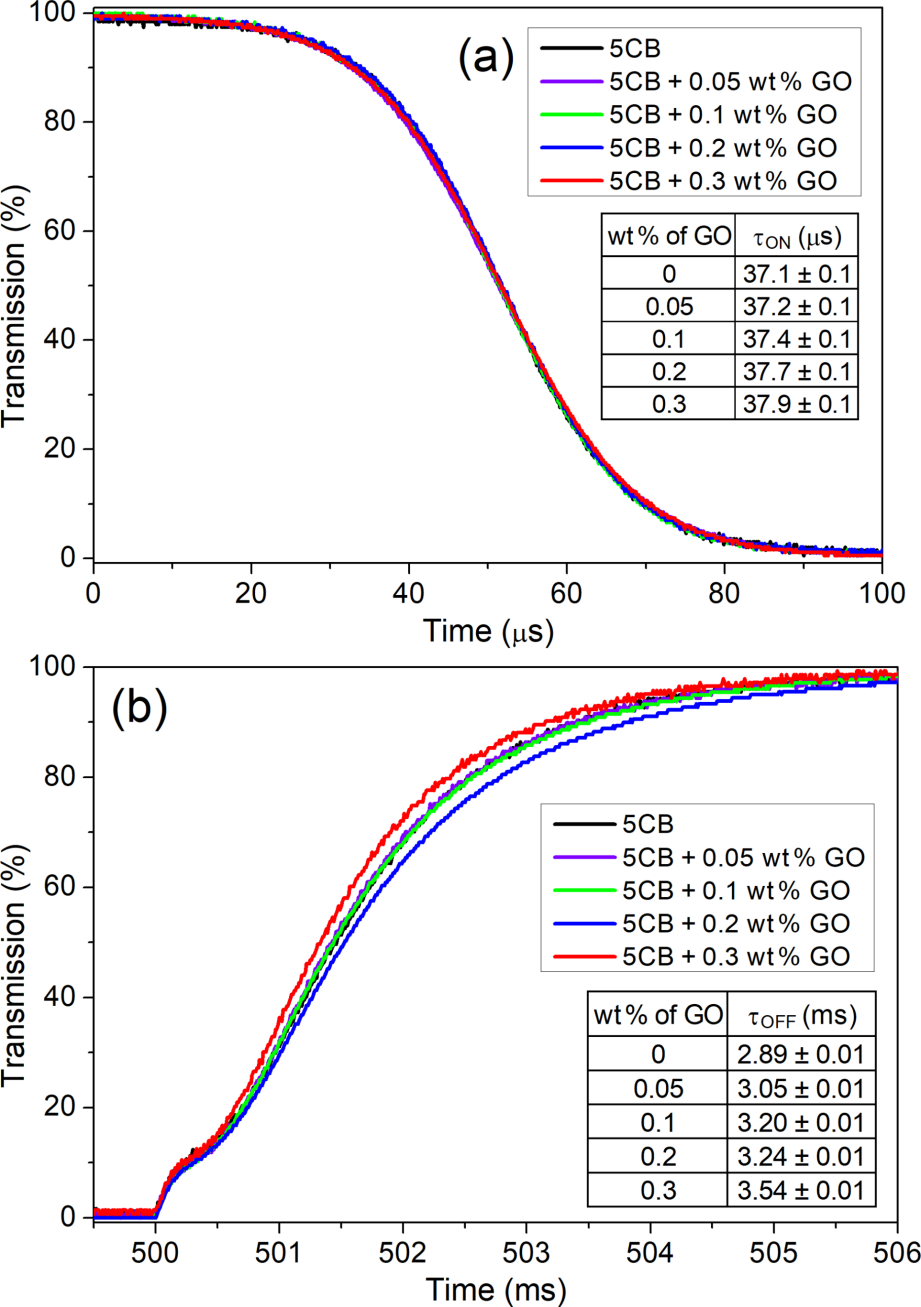
Electro-optical characteristics of the switching process in the cells with the 5CB-GO suspensions. Normalized light transmission as a function of time for (a) switch-on (τ_90–10_) and (b) switch-off (τ_10–90_) processes for different concentrations of GO flakes in the 5CB liquid crystal compound.

At high voltages (*U* > *U*_th_), the change of the director reorientation (*U* > *U*_th_) from planar to homeotropic alignment and vice versa is characterized by two parameters: the switch-on τ_ON_ and the switch-off τ_OFF_ time. The switch-on time depends on the magnitude of the applied voltage *U* as follows: τ_ON_ = γ*d*^2^/(ε_0_Δε*U*^2^ − π^2^*K*_11_), where γ is the rotational viscosity, *d* is the thickness of the cell and ε_0_ is the vacuum permittivity. In contrast, the switch-off time is determined mainly by splay elastic constant *K*_11_, τ_OFF_ = γ*d*^2^/(π^2^*K*_11_). After removing the electric field, the director ***n*** relaxes to the initial state due to the elastic nature of the medium. Figure 7 present the dynamic response of the liquid crystal cells filled with different GO suspensions. When the voltage (*U* = 10 V) is turned on, the transmission intensity decreases over time (Figure 7a). The addition of the GO flakes to 5CB does not change the optical response of the cell. Here, the switch-on time refers to the time between 90% to 10% transmission of light (τ_90–10_). It was found that τ_ON_ increases with the increase of the GO concentration. The effect can be attributed to the increase of the rotational viscosity γ. Figure 7b illustrates the τ_OFF_ dependence. The switch-off time is the time of the director relaxation after removing an electric signal at a given frequency. In the electro-optical characteristic is the time interval where the normalized transmission intensity at the level of 10% rises to 90% (τ_10–90_). The corresponding figures clearly depict that the flakes suspended in the nematic host recover to their initial state with different times, slower than the pure nematic liquid crystal, which is caused by the increase of the rotational viscosity due to the presence of the GO flakes.

**Figure 8:**
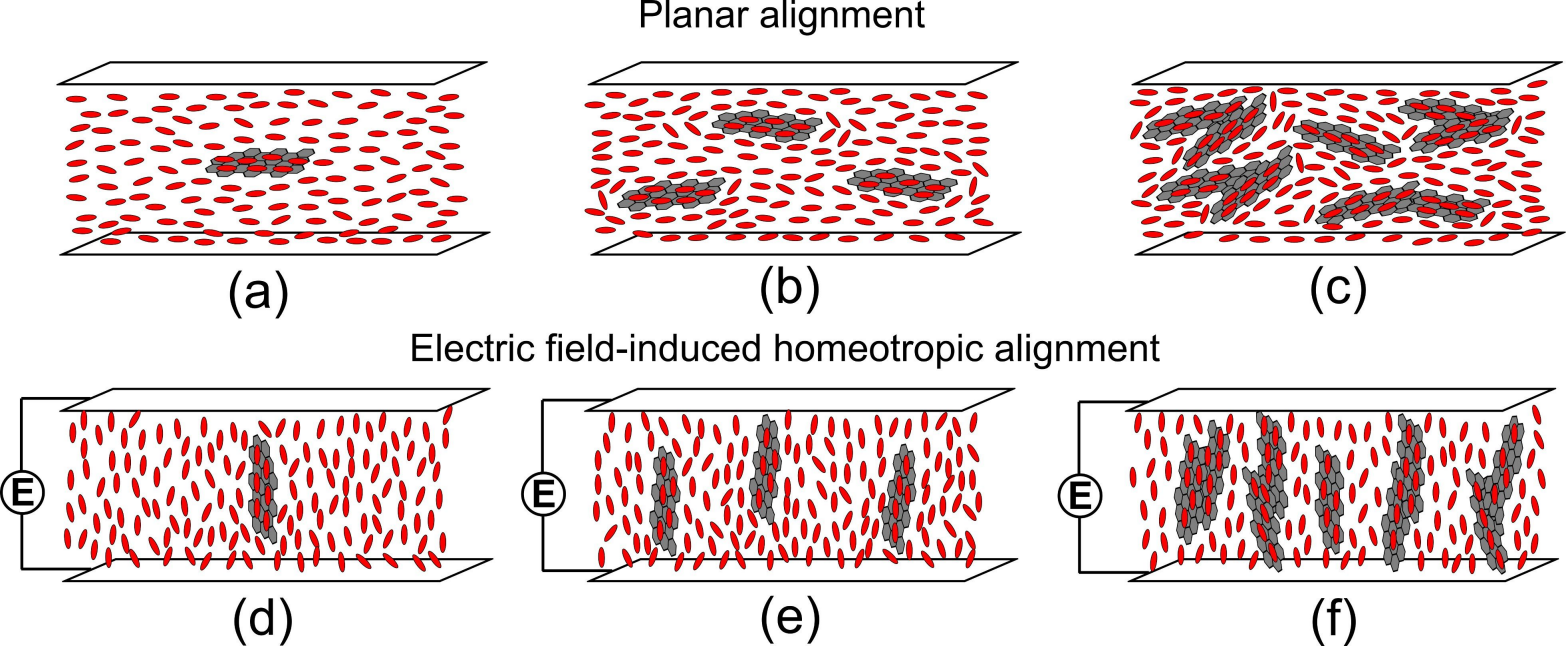
Liquid crystal cell filled with graphene oxide (GO) flakes suspended in a thermotropic nematic liquid crystal. The picture presents the nematic order in a planar oriented cell at (a) low, (b) medium, and (c) high concentrations of GO flakes and an electric-field-induced homeotropic alignment in the NLC-GO composite of (d) low, (e) medium, and (f) high concentration.

In the planar oriented cell without the electric field, the GO flakes are suspended parallel to the nematic director ***n***. At lower concentrations (0.05–0.1 wt %), the nematic order is increased around the GO sheets because of the π–π stacking between the liquid crystal molecules and the GO flakes (Figure 8a). GO flakes stiffen the structure. As the GO ratio increases, the strong π–π stacking induces the deformation of the director field (Figure 8b,c). Moreover, the rotational viscosity increases. At a concentration of 0.3 wt %, the GO aggregates disturb the nematic order significantly. The electric field induces the GO flake/nematic molecule reorientation process. Therefore, in the field on-state, the liquid crystal molecules with GO flakes reorient simultaneously in the field direction (Figure 8d–f). We assume that the electric field destroys the GO aggregates. After turning the electric field off, the distorted director does not accelerate the switching process back to the original orientation. When the electric field is in the off-state, the GO flakes follow the director due to the strong anchoring energy between 5CB’s benzene rings and the graphene’s honeycomb structure. The interactions between the liquid crystal molecules and the GO flakes are responsible for the lower elastic torque.

## Conclusion

We studied the change of the threshold voltage in dielectric spectroscopy and electro-optical measurements of the 5CB nematic liquid crystal with various concentrations of GO flakes (0.05–0.3 wt %). The effect of GO was significant. The reduction of the threshold voltage in the thin liquid crystal cell at the concentration of 0.2 wt % is related to the effects which can be interpreted as the decrease of the elastic constants. In our opinion the reason is related to the alignment of 5CB with respect to the 0.2 wt % GO flakes. When we added more GO flakes, the molecules of 5CB were not aligned properly and the threshold voltage was reduced. It was easier to trigger reorientation of the liquid crystal director when the structure was disrupted as compared to the pure 5CB. Of course, this imperfection in alignment of 5CB molecules is due to the π–π stacking between the 5CB’s benzene rings and the graphene oxide’s structure. At a low concentration (0.05 wt %) we observed the opposite effect. The structure was stiffened due to the strong interaction of NLC-GO. However, at a higher concentration (0.3 wt %) we noticed the reduction of the threshold voltage because of the significant increase of the rotational viscosity. Nematic liquid crystals with dispersed graphene oxide flakes are interesting candidates for potential applications in liquid crystal modulators and filters, smart windows, and isotropic liquid crystal displays.

## References

[1] Li B-X, Borshch V, Shiyanovskii S V, Liu S-B, Lavrentovich O D (2014). Appl Phys Lett.

[2] Li B-X, Borshch V, Shiyanovskii S V, Liu S-B, Lavrentovich O D (2015). Phys Rev E.

[3] Fréedericksz V, Zolina V (1933). Trans Faraday Soc.

[4] Yang D-K, Wu S-T (2006). Fundamentals of Liquid Crystal Devices.

[5] Hsu C-J, Lin L-J, Huang M-K, Huang C-Y (2017). Crystals.

[6] Haraguchi F, Inoue K, Toshima N, Kobayashi S, Takatoh K (2007). Jpn J Appl Phys, Part 2.

[7] Ha Y-S, Kim H-J, Park H-G, Seo D-S (2012). Opt Express.

[8] Sharma M, Sinha A, Shenoy M R (2015). Opt Mater.

[9] Reznikov Y, Buchnev O, Tereshchenko O, Reshetnyak V, Glushchenko A, West J (2003). Appl Phys Lett.

[10] Imamaliyev A R, Ramazanov M A, Humbatov S A (2018). Beilstein J Nanotechnol.

[11] Lagerwall J P F, Scalia G (2008). J Mater Chem.

[12] Javadian S, Dalir N, Kakemam J (2017). Liq Cryst.

[13] Dalir N, Javadian S (2018). J Appl Phys.

[14] Dalir N, Javadian S, Kakemam J, Yousefi A (2018). J Mol Liq.

[15] Basu R (2013). Appl Phys Lett.

[16] Basu R, Garvey A, Kinnamon D (2015). J Appl Phys.

[17] Chen H-Y, Lee W, Clark N A (2007). Appl Phys Lett.

[18] Basu R, Iannacchione G S (2009). Appl Phys Lett.

[19] Lee W, Wang C-Y, Shih Y-C (2004). Appl Phys Lett.

[20] Huang C-Y, Hu C-Y, Pan H-C, Lo K-Y (2005). Jpn J Appl Phys, Part 1.

[21] Huang C-Y, Pan H-C, Hsieh C-T (2006). Jpn J Appl Phys, Part 1.

[22] Narayan R, Kim J E, Kim J Y, Lee K E, Kim S O (2016). Adv Mater.

[23] Lin F, Tong X, Wang Y, Bao J, Wang Z M (2015). Nanoscale Res Lett.

[24] Kim J E, Han T H, Lee S H, Kim J Y, Ahn C W, Yun J M, Kim S O (2011). Angew Chem, Int Ed.

[25] Pei S, Cheng H-M (2012). Carbon.

[26] Dan B, Behabtu N, Martinez A, Evans J S, Kosynkin D V, Tour J M, Pasquali M, Smalyukh I I (2011). Soft Matter.

[27] Tie W, Bhattacharyya S S, Gao Y, Zheng Z, Shin E J, Kim T H, Kim M, Lee J H, Lee S H (2017). Nanomaterials.

[28] Tie W, Bhattacharyya S S, Lim Y J, Lee S W, Lee T H, Lee Y H, Lee S H (2013). Opt Express.

[29] Hummers W S, Offeman R E (1958). J Am Chem Soc.

[30] Boniecki M, Gołębiewski P, Wesołowski W, Woluntarski M, Piątkowska A, Romaniec M, Ciepielewski P, Krzyżak K (2017). Ceram Int.

[31] Dulinska-Molak I, Chlanda A, Li J, Wang X, Bystrzejewski M, Kawazoe N, Chen G, Swieszkowski W (2018). Micron.

[32] Zgłobicka I, Chlanda A, Woźniak M, Łojkowski M, Szoszkiewicz R, Mazurkiewicz-Pawlicka M, Święszkowski W, Wyroba E, Kurzydłowski K J (2017). J Phycol.

[33] Chlanda A, Kijeńska E, Rinoldi C, Tarnowski M, Wierzchoń T, Swieszkowski W (2018). Micron.

[34] Urban S, Gestblom B, Dabrowski R (1999). Phys Chem Chem Phys.

[35] Bogi A, Faetti S (2001). Liq Cryst.

[36] Mrukiewicz M, Perkowski P, Mazur R, Chojnowska O, Piecek W, Dąbrowski R (2016). J Mol Liq.

[37] Hadjichristov G B, Marinov Y G, Petrov A G, Marino L, Scaramuzza N (2016). J Phys: Conf Ser.

[38] Miller D S, Carlton R J, Mushenheim P C, Abbott N L (2013). Langmuir.

[39] Al-Zangana S, Iliut M, Turner M, Vijayaraghavan A, Dierking I (2016). Adv Opt Mater.

[40] Al-Zangana S, Iliut M, Boran G, Turner M, Vijayaraghavan A, Dierking I (2016). Sci Rep.

[41] Burylov S V, Zakhlevnykh A N (2013). Phys Rev E.

[42] Petrescu E, Cirtoaje C (2018). Beilstein J Nanotechnol.

